# Prognostic relevance of urokinase plasminogen activator detection in micrometastatic cells in the bone marrow of patients with primary breast cancer.

**DOI:** 10.1038/bjc.1997.467

**Published:** 1997

**Authors:** E. F. Solomayer, I. J. Diel, D. Wallwiener, S. Bode, G. Meyberg, M. Sillem, C. Gollan, M. D. Kramer, U. Krainick, G. Bastert

**Affiliations:** Department of Obstetrics and Gynaecology, University of Heidelberg, Germany.

## Abstract

**Images:**


British Journal of Cancer (1997) 76(6), 812–818
© 1997 Cancer Research Campaign

### Prognostic relevance of urokinase plasminogen activator detection in micrometastatic cells in the bone marrow of patients with primary breast cancer

E-F Solomayer[1], IJ Diel[1], D Wallwiener[1], S Bode[1], G Meyberg[1], M Sillem[1], Ch Gollan[1], MD Kramer[2], U Krainick[1] and G Bastert[1]

[1]Department of Obstetrics and Gynaecology and [2]Institute of Immunology, Laboratory for Immunopathology, University of Heidelberg, Germany

**Summary** Patients with an elevated level of urokinase plasminogen activator (uPA) in breast cancer tissue have an adverse prognosis. This study evaluated the prognostic relevance of uPA detection in disseminated tumour cells in bone marrow. Bone marrow was sampled intraoperatively from both iliac crests in 280 patients with primary breast cancer. Interphase cells were enhanced and stained immunocytologically with two antibodies: 2E11, which detects TAG 12 – a tumour-associated glycoprotein typically expressed by almost all breast cancer cells – and the anti-uPA antibody HD-UK9. Thirty-five of the 2E11-positive women ($n$ = 132, 47%) developed metastatic disease (median follow-up time 44 months). Of these, most were uPA positive ($n$ = 23, 65%) and only 12 were uPA negative. Patients with uPA-positive cells in bone marrow ($n$ = 98, 35%) had a significantly shorter metastasis-free interval (36 months) than women who were uPA negative (44.5 months). The worst prognosis was seen in patients positive for both markers (29.5 months), followed by those who were uPA negative and 2E11 positive (37 months). The detection of uPA on disseminated tumour cells characterizes a subgroup of patients with an even worse prognosis, who should undergo more aggressive adjuvant systemic therapy. For the first time, it was possible to evaluate an important qualitative parameter involved in the process of breast cancer metastases.

**Keywords:** tumour cell detection; urokinase plasminogen activator (uPA); breast cancer; micrometastasis; prognostic factor

Metastasis in breast cancer is a highly complex and hitherto poorly understood process. Tumour cells disseminate directly through the vascular and lymphatic system. Such circulating tumour cells can be best detected in bone marrow, in which they find favourable conditions for proliferation (Cote et al, 1991; Diel et al, 1994). The majority of these disseminated tumour cells disappear from the bone marrow sinusoids without initiating metastasis formation. However, some micrometastatic cells possess the prerequisites for invasion and sooner (before diagnosis) or later (up to 20 years after primary treatment) lead to development of metastatic disease. Thus, tumour cell shedding is an important step in the process of metastasis development but is not sufficient in itself (Orr et al, 1993). Circulating tumour cells have to possess certain qualities to cause metastasis. The clinical and pathophysiological relevance of such qualities that carry the potential for metastasis is as yet unknown. In the fascinating process of metastasis, many researchers have focused on proteases and adhesion molecules (uPA, cathepsin D, integrins, E-cadherin) (Needham et al, 1988; Duffy et al, 1990). The uPA system is one of the most extensively investigated families of proteases that are secreted in tumour tissue. The present study, however, evaluates the role of this system in the penultimate step of metastasis development.

Plasminogen activators (PAs) are serine proteases that catalyse the cleavage of the inactive proenzyme plasminogen to the active form plasmin (Jänicke et al, 1990; Mayer, 1990). PAs are produced by many cells, including tumour cells (Schmitt et al, 1992). Tumour stroma consists of extracellular matrix and a basal membrane. The proteolytic activity of the tumour cell, in which the uPA system plays the central role, is able to degrade this stroma and thus enable tumour cells to migrate. The median levels of urokinase plasminogen activator (uPA) and its inhibitors (PAI-1 and PAI-2) are significantly higher in malignant than in benign tumours (Foucre et al, 1991). Several studies have reported poor prognosis in women with uPA-positive and/or PAI-1-positive tumours compared with patients with uPA- and PAI-1-negative carcinomas. In patients with node-negative breast cancer, an increase in uPA in tumour cytosol seems to have the highest clinical relevance as an independent prognostic factor (Jänicke et al, 1993; Duffy et al, 1994). This suggests that this system plays a central role in the process of invasion and metastasis (Duffy et al, 1990; Schmitt et al, 1990; Foekens et al, 1994; Duggan et al, 1995). Prognostic significance in breast and other cancer types has also been recently ascribed to the receptor for uPA (uPAR), an important molecule in plasmin-mediated extracellular matrix degradation (Ganesh et al, 1994; Pedersen et al, 1994; Duggan et al, 1995; Grondahl-Hansen et al, 1995). Heiss et al (1995*a*) have found a correlation between uPAR expression in disseminated tumour cells and clinical prognosis in gastric cancer.

The uPA concentration in the primary tumour does not correlate with the tumour size nor the nodal status (Grondahl-Hansen et al, 1993; Jänicke et al, 1993; Foekens et al, 1994). Therefore, the involvement of the lymph nodes, but not the development of distant metastases, seems to be independent of the uPA concentration. The uPA expression of breast cancer cells disseminated to the bone marrow and its relevance for the development of metastasis has not yet been investigated.

*Received 28 November 1996*
*Revised 25 February 1997*
*Accepted 5 March 1997*

*Correspondence to:* E-F Solomayer, University of Heidelberg, Department of Obstetrics and Gynaecology, Voss-Str. 9, D-69115 Heidelberg, Germany

The aim of this study was to analyse the prognostic value of uPA-positive tumour cells in bone marrow. Therefore, smears of all patients were stained immunocytochemically with 2E11 and with anti-uPA monoclonal antibodies in a parallel staining. The results of both examinations were compared and evaluated.

### MATERIALS AND METHODS

Between 1989 and 1992, a total of 280 patients undergoing therapy for primary breast cancer at the Department of Obstetrics and Gynaecology of the University of Heidelberg were enrolled in this study. In these patients, 20 ml of bone marrow was aspirated from each anterior iliac crest, according to the Jamshidi technique (Jamshidi et al, 1980). The procedure was performed immediately after surgery while the patient was still under anaesthesia. Informed consent was obtained from all participants. The heparinized bone marrow was processed within 24 h. The bone marrow was differentially centrifuged across a Ficoll gradient (Ficoll/Hypaque, Biochrom Berlin; density 1.077 g $mol^{-1}$). Interphase cells were washed twice and resuspended with DMEM (Dulbecco's modified Eagle medium). Subsequently, 10 µl of the cell suspension was smeared onto slides. The slides were air dried and stored at –20°C. The tumour cells were incubated with the two antibodies 2E11 and HD-UK9 (parallel staining).

### Incubation with antibody 2E11

The monoclonal antibody 2E11 (also called BM2) binds to the tumour-associated glycoprotein TAG 12, a polymorphic epithelial mucin expressed in 97% of all breast cancers. 2E11 binds to the amino acid sequence 20–24 (A, P, D, T, R) of the mucin peptide and reacts with high-molecular-weight mucin double bands in the region of 400 kDa in Western immunoblotting. In addition to breast cancer tissue, 2E11 strongly stains the cytoplasm of ovarian and endometrial carcinoma. In contrast, no staining is seen in mesenchymal tissues and bone marrow (Bastert et al, 1987; Werner et al, 1988; Kaul et al, 1989). Before staining, the cells were fixed with 100% methanol. After blocking endogenous phosphatase activity with 20% acetic acid, 2.28% periodic acid and 2% laevamisole, the smears were incubated with the biotinylated form of the antibody 2E11 (stock solution 2 mg $ml^{-1}$; dilution 1:1000 with phosphate-buffered saline containing 1% bovine serum) for 1 h at room temperature. Immune complexes were made visible by

**Figure 1** Breast cancer cells in bone marrow stained with the anti-uPA monoclonal antibody

**Table 1** Characteristics of patients with respect to tumour cell detection (TCD) and detection of uPA-positive cells in bone marrow

| Prognostic factor | *n* | Tumour cell detection | | | *P*[a] | uPA | | | *P*[a] |
|---|---|---|---|---|---|---|---|---|---|
| | | Positive | | Negative No. | | Positive | | Negative No. | |
| | | No. | (%) | | | No. | (%) | | |
| Tumour size | | | | | | | | | |
| T1 | 106 | 35 | (33) | 71 | | 19 | (18) | 87 | |
| T2 | 121 | 53 | (44) | 68 | < 0.001 | 38 | (31) | 83 | < 0.001 |
| T3 | 23 | 17 | (74) | 6 | | 17 | (74) | 6 | |
| T4 | 30 | 27 | (90) | 3 | | 24 | (80) | 6 | |
| Nodal status | | | | | | | | | |
| N0 | 148 | 48 | (32) | 100 | < 0.001 | 31 | (21) | 117 | < 0.001 |
| N + | 132 | 84 | (64) | 48 | | 67 | (51) | 64 | |
| Receptors (*n* = 248) | | | | | | | | | |
| ER positive[b] | 169 | 84 | (50) | 85 | 0.8 | 59 | (35) | 110 | 0.31 |
| PR positive[b] | 146 | 65 | (44) | 81 | 0.02 | 55 | (38) | 91 | 0.98 |
| ER and PR negative | 90 | 41 | (45) | 49 | | 31 | (34) | 59 | |
| Menopausal status | | | | | | | | | |
| Pre | 102 | 37 | (36) | 65 | 0.02 | 30 | (29) | 110 | 0.34 |
| Post | 178 | 95 | (53) | 83 | | 68 | (38) | 72 | |
| Grade (*n* = 240) | | | | | | | | | |
| I + II | 140 | 55 | (39) | 85 | 0.041 | 51 | (36) | 89 | 0.99 |
| III | 100 | 56 | (56) | 44 | | 35 | (35) | 65 | |
| S-phase (*n* = 180) | | | | | | | | | |
| < 5% | 108 | 52 | (48) | 56 | 0.8 | 35 | (32) | 73 | 0.14 |
| ≥ 5% | 72 | 39 | (52) | 33 | | 31 | (43) | 41 | |
| TCD/uPA | 280 | 132 | (47) | 148 | | 98 | (35) | 182 | |

[a]Chi-square test. [b]ER, oestrogen receptor; PR, progesterone receptor; positive ≥ 20 fmol $mg^{-1}$ protein.

© Cancer Research Campaign 1997

use of avidin–biotin–alkaline phosphatase complexes (ABC test; Vectastain, Camon, Wiesbaden, Germany) and new fuchsin as the substrate. The analysis of two smears was defined positive if one or more 2E11-positive tumour cells were detected.

### Incubation with anti-uPA antibodies (HD-UK9)

In the present study, we used the antibody HD-UK9, which was raised against high-molecular-weight uPA and recognizes the B-chain of uPA. Its features have previously been described (Kramer et al, 1992). The antibody was used as a non-purified serum-free culture supernatant containing 10 μg of antibody per ml. The clone HD-UK9 was particularly suitable for our study because of its high affinity and specificity. Reaction with uPA-expressing macrophages and monocytes in bone marrow was observed. These cells were recognized morphologically.

The staining with HD-UK9 antibody was similar to the staining method described in the section entitled 'Incubation with antibody 2E11'. As this uPA antibody is not available in a biotinylated form, the blocking of endogenous phosphatase activity was performed using a commercially available kit (Dako APAAP-Kit, Dakopatts, Hamburg), which included a rabbit anti-mouse antibody as secondary antibody and Fast red for visualization (Kramer et al, 1992). The membrane and cytoplasm of the uPA-positive cells stained bright red (Figure 1). One negative and one positive smear were used as controls for all stains. Four smears were analysed per patient. The membrane and cytoplasm of the tumour cells stained bright red. The analysis of two smears was defined positive if one or more uPA-positive tumour cells were detected.

### Surgical and systemic adjuvant treatment

Surgical treatment was done either by mastectomy and axillary lymph node dissection ($n$ = 103) or by breast-conserving therapy, lymph node dissection and radiotherapy ($n$ = 177). The median number of histologically examined lymph nodes was 20. All patients with positive lymph nodes and those with negative lymph nodes but with other poor prognostic criteria (e.g. GIII, progesterone receptor status negative, S-phase fraction > 5%) received systemic adjuvant treatment. Detection of uPA and tumour cells in bone marrow was not taken into account when making therapeutic decisions. Patients were treated with tamoxifen (30 mg per day) for 2 years ($n$ = 87), six cycles of standard chemotherapy with cyclophosphamide (600 mg $m^{-2}$) + methotrexate (40 mg $m^{-2}$) + 5-fluorouracil (600 mg $m^{-2}$) ($n$ = 72), six cycles of cyclophosphamide (600 mg $m^{-2}$) + epirubicin (60 mg $m^{-2}$) ± 5-fluorouracil (600 mg $m^{-2}$) ($n$ = 10) or 3.6 mg of goserelin monthly for 2 years ($n$ = 15). Eighty-eight patients without axillary lymph node metastases and eight patients with one or two positive lymph nodes and good prognostic criteria received no further systemic treatment.

### Follow-up

All patients were seen according to the usual follow-up guidelines in the Oncology Outpatients Clinic at the Department of Obstetrics and Gynaecology of the University of Heidelberg. Follow-up intervals were adjusted according to the risk situation of the patients and ranged between 3 and 12 months. At each follow-up visit, a clinical examination was performed and the following laboratory parameters were evaluated: full blood count, erythrocyte sedimentation rate, tumour markers CEA and CA 15-3. Abdominal ultrasound scans (liver) and chest radiography were performed at least once yearly during the first 5 years and then at longer intervals. Bone scans were performed yearly during the first 2 years and according to clinical symptoms thereafter. If necessary, suspicious parts of the skeleton were radiographed.

### Statistics

The detection of 2E11-positive and uPA-positive cells in bone marrow were correlated to established prognostic factors using the chi-square test. All survival curves were calculated according to Kaplan–Meier analysis, and the comparisons between two survival curves were performed using the log-rank test after Mantel and Breslow (Collett, 1994). The stepwise Cox regression analysis was used to demonstrate the independence of prognostic factors (e.g. detection of 2E11-positive and uPA-positive cells in bone marrow). The relevance of individual variables in the Cox regression model was characterized by calculation of the relative risk (RR) (Cox, 1972). All reported $P$-values are two-sided. The statistical analysis was performed using Systat software (Evanstone, IL, USA).

### RESULTS

### Description of patients

The characteristics of the patients are shown in Table 1. In 81% of patients, the primary tumour was stage T1 ($n$ = 106) or T2 ($n$ = 121); only 19% had carcinomas with diameters exceeding 5 cm ($n$ = 23) or stage T4 ($n$ = 30). One hundred and forty-eight women (53%) were node negative, 132 women had lymph node metastases (47%). Tumours were oestrogen receptor positive in 68% of patients and progesterone receptor positive in 59%, while both receptors were negative in 36%. A total of 178 women were post-menopausal (63.6%) and 102 were premenopausal (36.4%). The tumours were graded as follows: grade I, 10 cases (4%); grade II, 130 cases (54%); and grade III, 100 cases (42%). The S-phase fraction of the tumour was above 5% in 72 women and below 5% in 108 women (60%). In some patients, values for S-phase ($n$ = 100), receptors ($n$ = 32) and grading ($n$ = 40) were not available.

In 132 patients, the tumour cells were positive for 2E11 (47%) and in 98 patients the tumour cells stained positively for uPA (35%).

### Correlation between established factors and 2E11 positivity

Table 1 shows the correlation calculations between 2E11 positivity and established prognostic factors. The correlation between 2E11, tumour size and nodal status was highly significant. Furthermore, the correlations between 2E11 and progesterone receptor positivity ($P$ = 0.02), grading ($P$ = 0.041) and menopausal status ($P$ = 0.020) were significant. No significant correlation was found with respect to oestrogen receptor status and S-phase fraction.

### Correlation between uPA positivity and established factors

The calculations of correlation between classic prognostic factors and uPA positivity are shown in Table 1. Significant correlations were seen between uPA positivity and tumour size ($P$ < 0.001) and nodal status ($P$ < 0.001). No significant correlation was seen with respect to other established prognostic factors, such as

© Cancer Research Campaign 1997

**Figure 2** Distant disease-free survival (**A**) and overall survival (**B**) of patients with primary breast cancer according to presence or absence of uPA-positive tumour cells detected in bone marrow

**Figure 3** Distant disease-free survival (**A**) and overall survival (**B**) of patients with uPA-negative or uPA-positive cells in bone marrow according to presence or absence of micrometastatic tumour cells detected in bone marrow

**Table 2** Results of multivariate analysis (Cox regression analysis stratified with respect to adjuvant therapy)

| Variable | *P* | RR[a] | 95% CI[a] |
|---|---|---|---|
| *Metastasis-free survival* | | | |
| TCD | < 0.001 | 3.05 | 1.45–5.86 |
| (positive, negative) | | | |
| Grade | 0.004 | 2.94 | 1.59–5.45 |
| (I + I, III) | | | |
| Nodal status | 0.020 | 2.39 | 1.18–4.85 |
| ($N_0$, $N_{1-3}$, $N_{4-9}$, $N_{>9}$) | | | |
| uPA detection | 0.035 | 1.64 | 1.59–5.45 |
| (positive, negative) | | | |
| *Overall survival* | | | |
| TCD | 0.007 | 4.23 | 1.58–11.2 |
| (positive, negative) | | | |
| Progesterone receptor | 0.002 | 3.11 | 1.50–6.43 |
| (positive, negative[b]) | | | |
| Nodal status | < 0.001 | 2.24 | 1.23–5.69 |
| ($N_0$, $N_{1-3}$, $N_{4-9}$, $N_{>9}$) | | | |
| uPA detection | 0.019 | 1.8 | 1.62–5.87 |
| (positive, negative) | | | |

[a]Cox regression stratified by adjuvant therapy. Nodal status was included in the model as one variable with values 1, 2, 3 and 4 given to the groups as indicated. Relative risk (RR), therefore, refers to the comparison of one category to the next. CI, confidence interval; TCD, tumour cell detection (2E11); uPA, detection of urokinase plasminogen activator-positive cells. [b]Positive ≥ 20 fmol $mg^{-1}$ protein.

grading, S-phase fraction, oestrogen and progesterone receptor status and menopausal status.

### Follow-up

Distant metastases were detected in 67 patients after a median follow-up of 44 months. Twenty-four patients developed bone metastases, 27 visceral metastases (lung, $n$ = 14; liver, $n$ = 16; brain, $n$ = 3) and 16 both (osseous and visceral). Tumour cells (2E11 positivity) were detected in bone marrow smears from 53 women (79%). In 41 patients (61%), bone marrow cells reacted positively with the uPA antibody. Smears negative for antibody were found in only 11 patients (16%) with distant metastases. Thirty-eight of 53 smears (71%) were positive for tumour cells (2E11) as well as for uPA. In 15 women (29%) with 2E11-positive cells in bone marrow, the uPA stains were negative. Of the 14 2E11-negative patients with distant metastases, only three (21%) were positive for uPA.

Patients with 2E11-positive cells in bone marrow had a metastasis-free interval (MFI) of 33 months and an overall survival (OAS) of 37 months (median). Women in whom tumour cells were not detected in bone marrow had significantly longer MFIs (median 47 months, $P$ < 0.001) as well as significantly longer survival times (median 48.5 months). Women with uPA-positive cells in bone marrow had a significantly shorter MFI (median 36 months) compared with patients with uPA-negative bone marrow (median 44.5 months; $P$ < 0.001) (Figure 2A and B). Similarly, the survival time was significantly shorter (40 vs 45; $P$ < 0.001; Figure 2A and B). If the results for both antibodies were combined, patients with positive reactions for both 2E11 and uPA had the worst prognosis (MFI 29.5 months; OAS 34 months) followed by the group with positive 2E11 but negative uPA (MFI 37 months; OAS 38 months). The best prognosis was found in women who

© *Cancer Research Campaign 1997*

were negative for 2E11 (MFI 47 months; OAS 48.5 months) (Figure 3A and B).

Univariate analysis showed tumour cell detection with 2E11 ($P < 0.001$), nodal status ($P < 0.001$), grading ($P = 0.007$) and detection of uPA-positive tumour cells in bone marrow ($P = 0.001$) to be prognostic factors for the development of distant metastases. On the other hand, tumour size, S-phase fraction, oestrogen and progesterone receptor status, and menopausal status had no influence on the MFI. With respect to overall survival, tumour cell detection with 2E11, nodal status, detection of uPA-positive cells, progesterone receptor status and grading were significant.

We investigated whether positive uPA and 2E11 detection in bone marrow are strong independent prognostic factors by performing a multivariate analysis stratified with respect to adjuvant therapy (Cox regression). Both uPA and 2E11 were independent factors for distant disease-free survival [2E11: $P < 0.001$, relative risk (RR) 3.05, 95% confidence interval (CI) 1.45–5.86; uPA: $P = 0.35$, RR = 1.64, CI = 1.59–5.45]. With respect to the overall survival, tumour cell detection (2E11) in bone marrow reached the highest level of significance ($P = 0.007$, RR = 4.23, CI = 1.58–11.2). uPA detection in bone marrow was again identified as an independent prognostic factor ($P = 0.019$, RR = 1.8, CI = 1.62–5.87; Table 2).

### DISCUSSION

The development of distant metastases is decisive for the fate of patients with breast cancer. Although the axillary lymph node status is considered to be the best prognostic factor with regard to disease-free survival and overall survival, it reflects only indirectly the process of haematogenic metastasis. Therefore, 30% of patients with node-negative breast cancer at primary surgery will relapse within 10 years (Rosen et al, 1989). Tumour cell detection (TCD) in bone marrow can offer an alternative to the prognostic value of the nodal state. We have shown previously that TCD has greater prognostic relevance than the axillary lymph node status in a defined subgroup of patients with small tumours (Diel et al, 1996). However, the fate of disseminated cancer cells can be influenced by the qualitative properties of these cells and by several host factors (Heiss et al, 1995*a*). Studies on disseminated tumour cells in bone marrow offer the possibility of investigating the penultimate step in the development of metastasis and to gain information about the induction and regulation of the ensuing growth (Frassica and Sim, 1988; Pantel et al, 1993; Diel et al, 1994).

Only a few studies have analysed qualitative properties on micrometastatic tumour cells. Pantel et al (1993) investigated the expression of Ki-67, p120 and *Erb*B2 on micrometastatic breast and gastric cancer cells. They found a high incidence of *Erb*B2 expression on disseminated breast cancer cells in patients with metastasis. The authors postulate that these cells might have been positively selected during the early stages of metastasis. Riesenberg et al (1993) showed that micrometastatic prostatic cancer cells could express prostate-specific antigen. Recently, Heiss et al (1995*a*) reported that useful prognostic information can be obtained by analysis of the plasminogen activator (PA) system on disseminated gastric tumour cells. The authors showed a strong association between survival and the expression of uPAR on disseminated gastric cancer cells in bone marrow. Their results suggest the importance of investigating uPA on disseminated tumour cells.

The most widely investigated plasminogen activators in the prognosis of breast cancer are uPA, PAI-1, PAI-2 and uPAR. High uPA and PAI-1 levels are correlated with a poor prognosis for patients with primary breast cancer (Duffy et al, 1990; Jänicke et al, 1990, 1993; Foekens et al, 1992, 1994; Grondahl-Hansen, 1993). The PA system appears to be a strong prognostic marker in breast cancer as well as in other types of cancer (Nekarda et al, 1994; Pedersen et al, 1994; Cantero et al, 1997). Recently, the prognostic significance of the uPAR in breast cancer (Grondahl-Hansen, 1995), colorectal cancer (Ganesh et al, 1994), gastric cancer (Heiss et al, 1995*b*), pancreatic cancer (Cantero et al, 1997) and lung cancer (Pedersen et al, 1994) has been shown. While numerous studies have reported an increase in uPA expression during transmigration of basal membranes in extracellular matrix in metastatic and non-metastatic tumour cell lines, this has not previously been investigated in breast cancer cells that have already been shedded (Ossowski et al, 1991; Henderson et al, 1992). Here, proteolytic enzymes need to be activated to enable transmigration of the vascular endothelium and the perivasal matrix.

The objectives of this study were firstly to detect uPA in disseminated tumour cells as an indicator of increased potential for invasion and proliferation and, secondly, to compare the individual cellular characteristics with respect to their prognostic relevance at the time of primary surgery.

Tumour cell detection with 2E11 has been established in our clinic and is routinely used. The proportion of tumour cell-positive patients (positive reaction with 2E11) of 47% ($n = 132$) was in the same range as in our previous studies (Diel et al, 1992, 1994, 1996). TCD in bone marrow correlates with prognosis in patients with primary breast cancer. Other authors obtained similar results (Mansi et al, 1987; Schlimok et al, 1987; Untch et al, 1988; Osborne et al, 1991). The bone marrow of 80% of patients with subsequent metastasis was TCD positive at the time of first diagnosis (Diel et al, 1996). Previously, tumour cells in bone marrow have been characterized by immunocytological differences from autochthonous bone marrow (e.g. mucin antibodies, cytokeratin). For the first time, we describe a marker that characterizes the biological behaviour of the breast cancer cell and therefore may be of crucial relevance for the final steps involved in metastatic development.

In 35% of patients ($n = 98$), cells or cell clusters stained positively with a monoclonal antibody against uPA (HD-UK-9). Highly significant correlation with other prognostic factors was seen for tumour size and nodal status but not for any other factors. Patients with uPA-positive cells in bone marrow showed a significantly shorter MFI compared with women without uPA-positive cells in bone marrow ($P < 0.001$). Similar results were obtained with respect to overall survival ($P < 0.001$). Multivariate analysis showed uPA detection in bone marrow to be an independent prognostic factor (for both MFI and OAS).

The worst prognosis was seen in patients positive for both 2E11 and uPA, followed by patients with negative uPA and positive 2E11 staining (Figure 2 A and B, Figure 3 A and B). In 40% of patients positive for both 2E11 and uPA, distant metastases developed after a median of 44 months. Thus, the additional detection of uPA permits the definition of a subgroup with a very poor prognosis. This proves that tumour cell dissemination to the bone marrow is a necessary step in the process of metastasis development but is probably not sufficient in itself. Additionally, tumour cells have to possess certain biological qualities (Heiss et al, 1995*a*). In our study, we showed that cells that have left the capillary system in the target organ have a potential for invasive growth. uPA-positive cells possess a higher metastatic affinity than uPA-negative cells. It is likely that other secreted proteases, adhesion

 © Cancer Research Campaign 1997

molecules and angiogenic properties are also involved in the process of metastasis development.

The macrophage-mediated proteolytic activity is thought to be involved in the invasion and subsequent distant spreading of malignancy (Pyke et al, 1993). However, uPA is synthesized not only by stromal cells but also by the tumour cells themselves (Chistensen et al, 1996; Constantini et al, 1996). Although it may be anticipated that stromal cells and tumour cells cooperate with respect to the functioning of the plasminogen activator system (Bianchi et al, 1994), it may be expected that at least a fraction of the primary tumour cells may themselves express uPA and/or the uPAR. It is tempting to speculate that this subfraction may comprise particularly malignant cells of the primary tumour with an increased potential for metastasis formation (and increased probability appearing in the bone marrow).

The highly significant correlation between uPA-positive cells in bone marrow, tumour size, nodal status and 2E11 positivity suggests a possible role of these parameters in the process of haematogenic metastasis and invasion. Tumour cells were more frequently found in the bone marrow of patients with tumour size T3–4 and lymph node involvement. On the one hand, this might be due to the size of the primary tumour, which may lead to an increased shedding of tumour cells proportional to the growth of the tumour. This increases the probability of lymph node and distant metastasis. On the other hand, there was a remarkably high rate of uPA-positive tumour cells in the bone marrow of large and particularly nodal positive tumours. An association between uPA level in primary tumours and axillary lymph node involvement has not been found (Jänicke et al, 1990, 1993). It could be speculated that either uPA-positivity is acquired during the growth of the primary tumour or, conversely, that initially uPA-positive cells are able to enhance the local growth and the haematogenic metastasis but not the lymphogenic potential of the tumour.

We performed parallel staining with 2E11 and HD-UK9 antibodies. The bone marrow cells were characterized by two markers (expression of TAG 12 and uPA), which were determined on two different smears. We cannot be absolutely sure that uPA-positive cells are definitely 2E11 positive or vice versa. Generally, reactions of HD-UK9 with uPA-expressing macrophages and monocytes in bone marrow could be excluded by morphology. Typically, a deep red corona with numerous dark pericellular granules was seen. However, in ten cases, cells were stained that did not fulfil the criteria for malignancy and that were identified as macrophages or myeloid precursor cells (and were evaluated as negative for prognostic relevance). These disadvantages in our method (parallel staining) can be avoided by using the double-staining methods (Pantel et al, 1993; Riesenberg et al, 1993; Heiss et al, 1995*a*) that we now use after resolving technical problems.

Of the patients with distant metastasis and uPA-positive smears, all but three were positive in parallel stainings for 2E11 and were therefore tumour cell positive. UPA positivity in bone marrow was found to be an important prognostic factor in the 2E11-positive group but not in the 2E11-negative group. The results concerning the good prognostic value of uPA detection in bone marrow demonstrate that uPA was determined on or in association with tumour cells.

The worse prognosis of patients with uPA expression on micrometastatic cells in bone marrow could indicate the need for a more aggressive adjuvant therapy in this subgroup. In recurrent breast cancer, uPA and PAI-1 are predictors of poor response to tamoxifen therapy (Foekens et al, 1995). The question of whether the plasminogen activator system plays a predictive role for adjuvant therapy in a high-risk group can only be answered by further clinical trials.

We conclude that the expression of uPA characterizes a group of tumour cells that have not only detached from the primary tumour and succeeded in extravasation but that have an inherent potential for the development of clinically relevant metastasis. This is reflected by a shorter MFI and shorter overall survival in uPA-positive patients.

### REFERENCES

Bastert G, Eichler A and Kaul S (1987) Monoclonal antibodies against breast cancer. In *Immune Deficient Animals in Biomedical Research*, Rygaard K, Brünner N and Spang-Thomsen M. (eds), pp. 224–227. S Karger: Basle

Bianchi E, Cohen RL, Thor AT, Todd RF, Mizukami IF, Lawrence DA, Ljung BM, Shuman MA and Smith HS (1994) The urokinase receptor is expressed in invasive breast cancer but not in normal breast tissue. *Cancer Res* **54**: 861–866

Cantero D, Friess H, Deflorin J, Zimmermann A, Bründler M-A, Riesle E, Korc M and Büchler MW (1997) Enhanced expression of urokinase plasminogen activator and its receptor in pancreatic carcinoma. *Br J Cancer* **75**: 388–395

Christensen L, Wiborg-Simonsen AC, Heegaard CW, Moestrup SK, Anderson JA and Andreasen PA (1996) Immunohistochemical localisation of urokinase-type plasminogen activator, type-1 plasminogen activator inhibitor, urokinase receptor and α(2)macroglobulin receptor in human breast carcinomas. *Int J Cancer* **66**: 441–452

Collett D (1994) *Modelling Survival Data in Medical Research*. Chapman & Hall: London

Constantini V, Sidoni A, Deveglia R, Cattato OA, Welleza G, Ferri I, Bucciarelli E and Nenci GG (1996) Combined overexpression of urokinase, urokinase receptor, and plasminogen activator inhibitor 1 is associated with breast cancer regression: an immunohistochemical comparison of normal, begnign and malignant breast tissues. *Cancer* **77**: 1079–1088

Cote RJ, Rosen PP, Lesser ML, Old LJ and Osborne MP (1991) Prediction of early relapse in patients with operable breast cancer by detection of occult bone marrow micrometastases. *J Clin Oncol* **9**: 1749–1756

Cox DR (1972) Regression models and life-tables. *J R Stat Soc (B)* **34**: 187–220

Diel IJ, Kaufmann M, Goerner R, Costa SD, Kaul S and Bastert G (1992) Detection of tumor cells in bone marrow of patients with primary breast cancer: a prognostic factor for distant metastasis. *J Clin Oncol* **10**: 1534–1539

Diel IJ, Kaufmann M, Costa SD and Bastert G (1994) Monoclonal antibodies to detect breast cancer cells. In: *Important Advances in Oncology*. DeVita VT, Hellman S and Rosenberg SA. (eds), pp. 143–164. Lippincott: Philadelphia

Diel IJ, Kaufmann M, Costa SD, Holle R, Von Minckwitz G, Solomayer EF, Kaul S and Bastert G (1996) Breast cancer patients with micrometastatic tumor cells in bone marrow at primary surgery – prognostic impact in comparison to nodal status. *J Natl Cancer Inst* **88**: 1652–1658

Duffy MJ, Reilly D, O'Sullivan C, O'Higgins N, Fennely JJ and Andreasen P (1990) Urokinase plasminogen-activator, a new and independent prognostic marker in breast cancer. *Cancer Res* **50**: 6827–6829

Duffy MJ, Reilly D, McDermott E, O'Higgins N, Fennelly JJ and Andreasen PA (1994) Urokinase plasminogen activator as a prognostic marker in different subgroups of patients with breast cancer. *Cancer Res* **74**: 2276–2280

Duggan C, Maguire T, McDermott E, O'Higgins N, Fennelly JJ and Duffy MJ (1995) Urokinase plasminogen activator and urokinase plasminogen activator receptor in breast cancer. *Int J Cancer* **61**: 597–600

Foekens JA, Schmitt M, van Putten WL, Peters HA, Bontenbal M, Jänicke F and Klijn JG (1992) Prognostic value of urokinase-type plasminogen activator in 671 primary breast cancer patients. *Cancer Res* **52**: 6101–6105

Foekens JA, Schmitt M, van Putten WL, Peters HA, Kramer MD, Jänicke F and Klijn JGM (1994) Plasminogen activator inhibitor-1 and prognosis in primary breast cancer. *J Clin Oncol* **12**: 1648–1658

Foekens JA, Look MP, Peters HA, van-Putten WL, Portengen H and Klijn JG (1995) Urokinase-type plasminogen activator and its inhibitor PAI-1: predictors of poor response to tamoxifen therapy in recurrent breast cancer. *J Natl Cancer Inst* **87**: 751–756

Foucre D, Bouchet C, Hacene K, Pourreau-Schneider N, Gentile A, Martin PM, Desplaces A and Oglobine J (1991) Relationship between cathepsin D, urokinase, and plasminogen activator inhibitors in malignant vs benign breast tumors. *Br J Cancer* **64**: 926–932

© Cancer Research Campaign 1997

Frassica FJ and Sim FH (1988) Pathogenesis and prognosis. In: *Diagnosis and Management of Metastatic Bone Disease*. Sim FH. (ed.), p. 16. Raven Press: New York

Ganesh S, Sier CF, Heerding MM, Griffioen G, Lamers CB and Verspaget HW (1994) Urokinase receptor and colorectal cancer survival (letter). *Lancet* **344**: 401–402

Grondahl-Hansen J, Christensen IJ, Rosenquist C, Brunner N, Mouridsen HT, Dano K and Blichert-Toft M (1993) High levels of urokinase-type plasminogen activator and its inhibitor PAI-1 in cytosolic extracts of breast carcinomas are associated with poor prognosis. *Cancer Res* **53**: 2513–2521

Grondahl-Hansen J, Peters HA, van Putten WLJ, Look MP, Pappot H, Ronne E, Dano K, Klijn JGM, Brünner N and Foekens JA (1995) Prognostic significance of the receptor for urokinase plasminogen activator in breast cancer. *Clin Cancer Res* **1**: 1079–1087

Heiss MM, Allgayer H, Gruetzner KU, Funke I, Babic R, Jauch KW and Schildberg FW (1995*a*) Individual development and uPA-receptor expression of disseminated tumour cells in bone marrow: a reference to early systemic disease in solid cancer. *Nature Med* **1**: 1035–1039

Heiss MM, Babic R, Allgayer H, Gruetzner KU, Jauch KW, Loehrs U and Schildberg FW (1995*b*) Tumor-associated proteolysis and prognosis: new functional risk factors in gastric cancer defined by the urokinase-type plasminogen activator system. *J Clin Oncol* **13**: 2084–2093

Henderson BR, Tansey WP, Phillips SM, Ramshaw IA and Kefford RF (1992) Transcriptional and posttranscriptional activation of urokinase plasminogen activator gene expression in metastatic tumor cells. *Cancer Res* **52**: 2489–2496

Jänicke F, Schmitt M, Hafter R, Hollrieder A, Babic R, Ulm K, Gössner W and Graeff H (1990) Urokinase-type plasminogen activator (uPA) antigen is a predictor of early relapse in breast cancer. *Fibrinolysis* **4**: 69–78

Jänicke F, Schmitt M, Pache L, Ulm K, Harbeck N, Höfler H and Graeff H (1993) Urokinase (uPA) and its inhibitor PAI-1 are strong and independent prognostic factors in node-negative breast cancer. *Breast Cancer Res Treat* **24**: 195–208

Jamshidi K, Swaim WR and Raisz G (1980) A new trephine for closed bone marrow biopsy. *Acta Haemat* **64**: 216

Kaul S, Windecker S and Bastert G (1989) Monoclonal antibodies reactive with tumor-associated epitopes of breast mucin glycoproteins *Proc Am Assoc Cancer Res* **30**: 349

Kramer MD, Vettel U, Schmitt M, Reinartz J, Brunner G and Meissauer A (1992) Monoclonal antibodies against plasminogen activators and plasminogen. *Fibrinolysis* **6** (suppl. 4): 103–111

Mansi JL, Berger U, Easton D, McDonnell T, Redding WH, Gazet J-C, McKinna A, Powles TJ and Coombes RC (1987) Micrometastases in bone marrow in patients with primary breast cancer: evaluation as an early predictor of bone metastases. *Br Med J* **295**: 1093–1096

Mayer M (1990) Biochemical and biological aspects of the plasminogen activator system. *Clin Biochem* **23**: 197–211

Nekarda H, Schmitt M, Ulm K, Wenninger A, Vogelsang H, Becker K, Roder JD, Fink U and Siewert JR (1994) Prognostic impact of urokinase-type plasminogen activator and its inhibitor PAI-1 in completely resected gastric cancer. *Cancer Res* **54**: 2900–2907

Needham GK, Nicholson S, Angus B, Farndon JR and Harris AL (1988) Relationship of membrane-bound tissue type and urokinase type-plasminogen activators in human breast cancers to estrogen and epidermal growth factor receptors. *Cancer Res* **48**: 6603–6607

Orr FW, Kostenuik P, Sanchez-Sweatman OH and Singh G (1993) Mechanism involved in the metastasis of cancer to bone. *Breast Cancer Res Treat* **25**: 151–163

Osborne MP, Wong GY, Asina S, Old LJ, Cote RJ and Rosen PP (1991) Sensitivity of immunocytochemical detection of breast cancer cells in human bone marrow. *Cancer Res* **51**: 2706–2709

Ossowski L, Russo-Payne H and Wilson EL (1991) Inhibition of urokinase-type plasminogen activator by antibodies: the effect on dissemination of a human tumor in the nude mouse. *Cancer Res* **51**: 274–281

Pantel K, Schlimok G, Braun S, Kutter D, Lindemann F, Schaller G, Funke I, Izbicki JR and Riethmüller G (1993) Differential expression of proliferation-associated molecules in individual micrometastatic carcinoma cells. *J Natl Cancer Inst* **85**: 1419–1424

Pedersen H, Brünner N, Francis D, Osterlind K, Ronne E, Hausen HH, Dano K and Grondahl-Hansen J (1994) Prognostic impact of urokinase, urokinase receptor, and type 1 plasminogen activator inhibitor in squamous and large cell lung cancer tissue. *Cancer Res* **54**: 4671–4675

Pyke C, Graem N, Ralfkiaer E, Ronne E, Hoyer-Hansen G, Brunner N and Dano K (1993) Receptor for urokinase is present in tumor-associated macrophages in ductal breast carcinoma. *Cancer Res* **53**: 1911–1915

Riesenberg R, Oberneder R, Kriegmair M, Epp M, Bitzer U, Hofstetter A, Braun S, Riethmüller G and Pantel K (1993) Immunocytochemical double staining of cytokeratin and prostate specific antigen in individual prostatic tumour cells. *Histochemistry* **99**: 61–66

Rosen PR, Groshen S, Saigo PE, Kinne DW and Hellman S (1989) A long-term follow-up study of survival in stage I (T1N0M0) and stage II (T1N1M0) breast carcinoma. *J Clin Oncol* **7**: 355–366

Schlimok G, Funke I, Holzmann B, Göttlinger G, Schmidt G, Häuser H, Swierkot S, Warnecke HH, Schneider B, Koprowski H and Riethmüller G (1987) Micrometastatic cancer cells in bone marrow: in vitro detection with anti-cytokeratin and in vivo labeling with anti-17-1A monoclonal antibodies. *Proc Natl Acad Sci USA* **84**: 8672–8676

Schmitt M, Jänicke F and Graeff H (1990) Tumor-associated fibrinolysis: the prognostic relevance of plasminogen activators uPA and tPA in human breast cancer. *Blood Coagul Fibrinolysis* **1**: 695–702

Schmitt M, Jänicke F, Moniwa N, Chucholowski N, Pache L and Graeff H (1992) Tumor-associated urokinase-type plasminogen activator: biological and clinical significance. *Biol Chem Hoppe Seyler* **373**: 611–622

Untch M, Harbeck N and Eiermann W (1988) Micrometastases in bone marrow in patients with breast cancer (letter). *Br Med J* **296**: 290

Werner M, Kaul S and Bastert G (1988) Biochemical characterization of the mucin like tumor associated antigen TAG-12. *J Cancer Res Clin Oncol* **114**: 59

© Cancer Research Campaign 1997


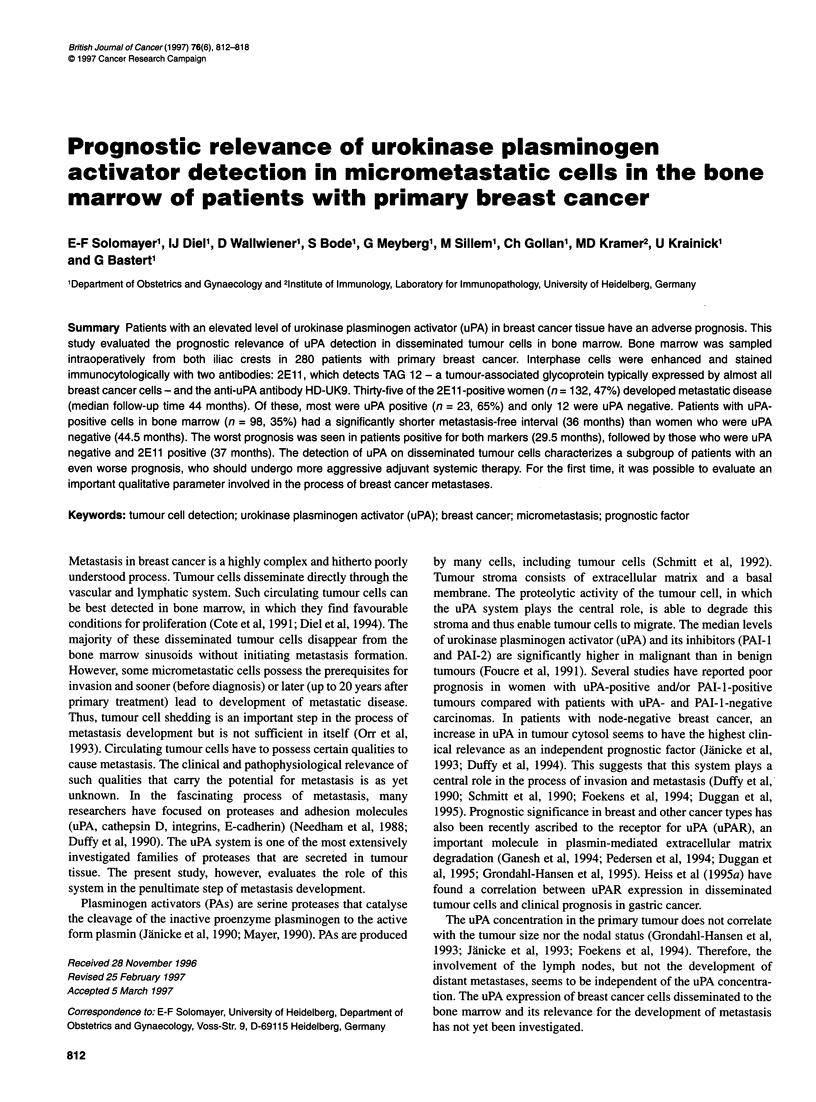

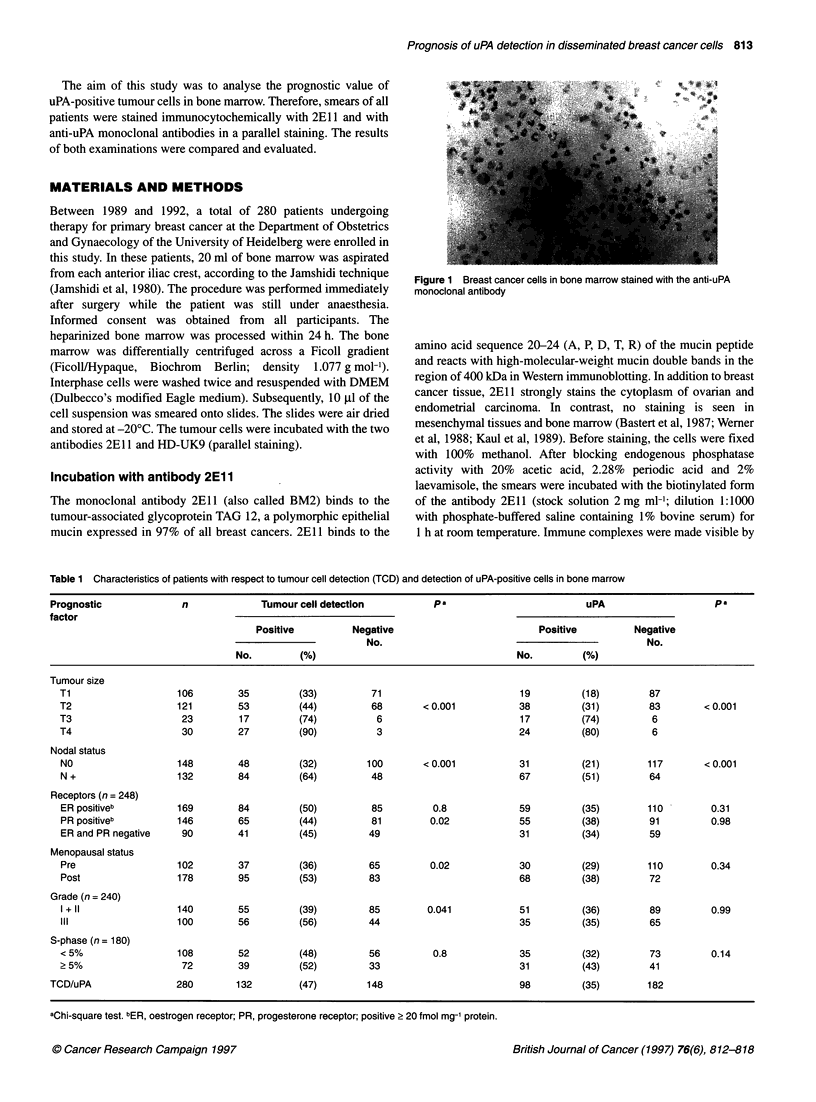

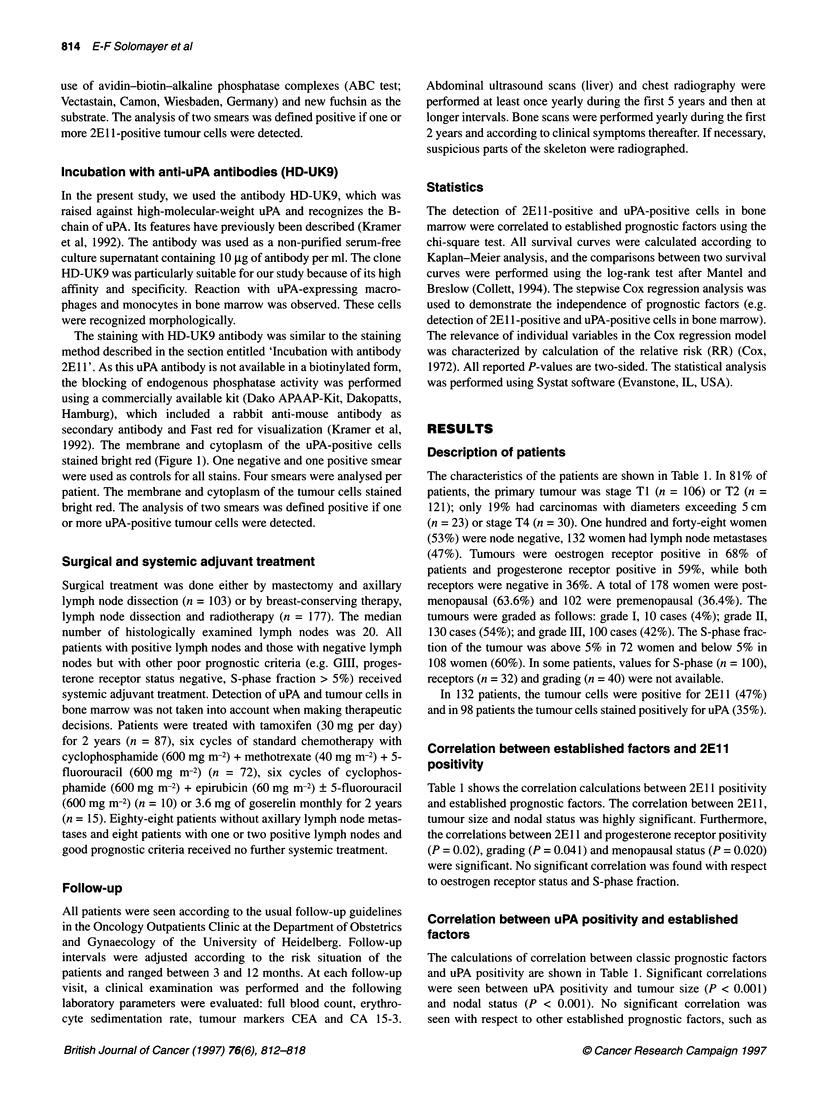

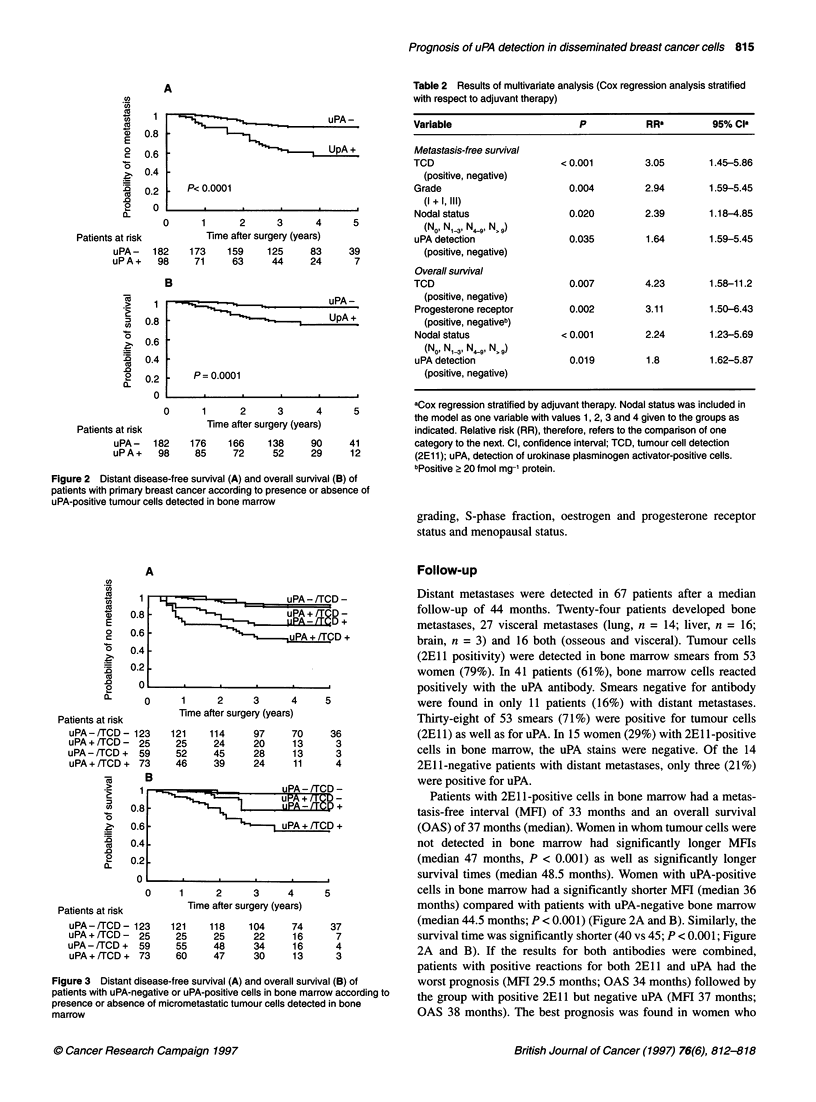

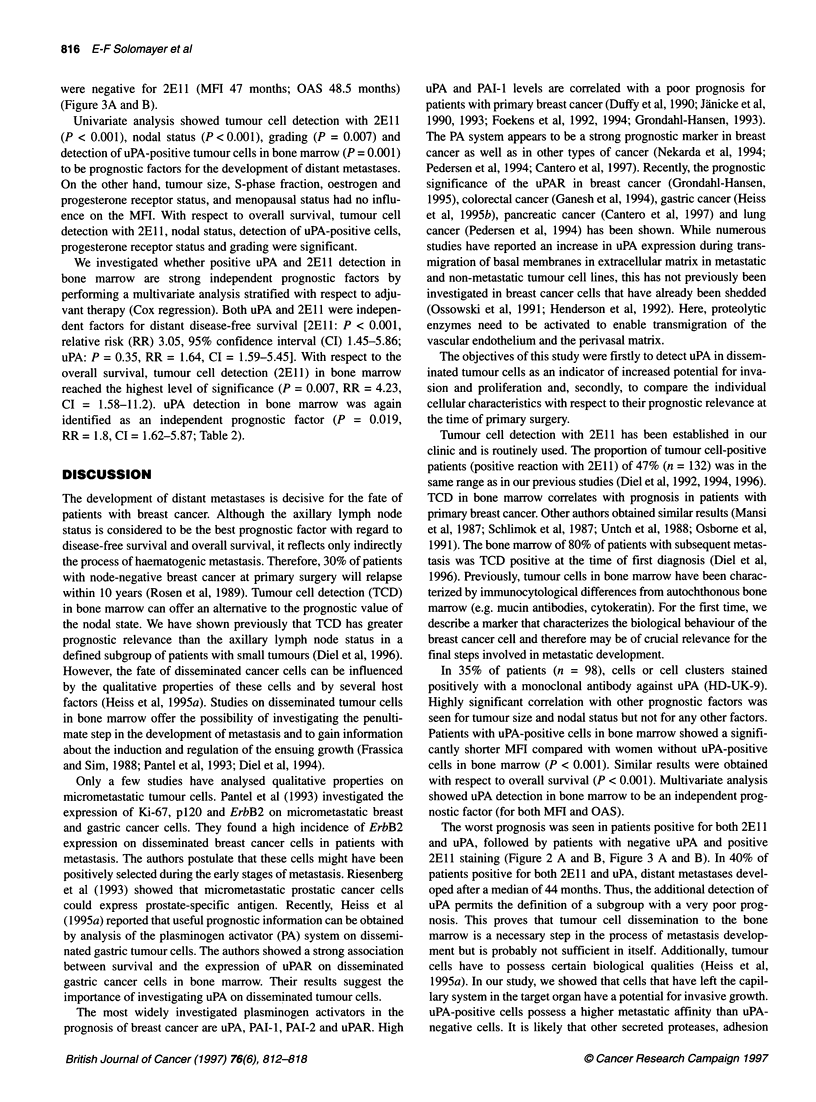

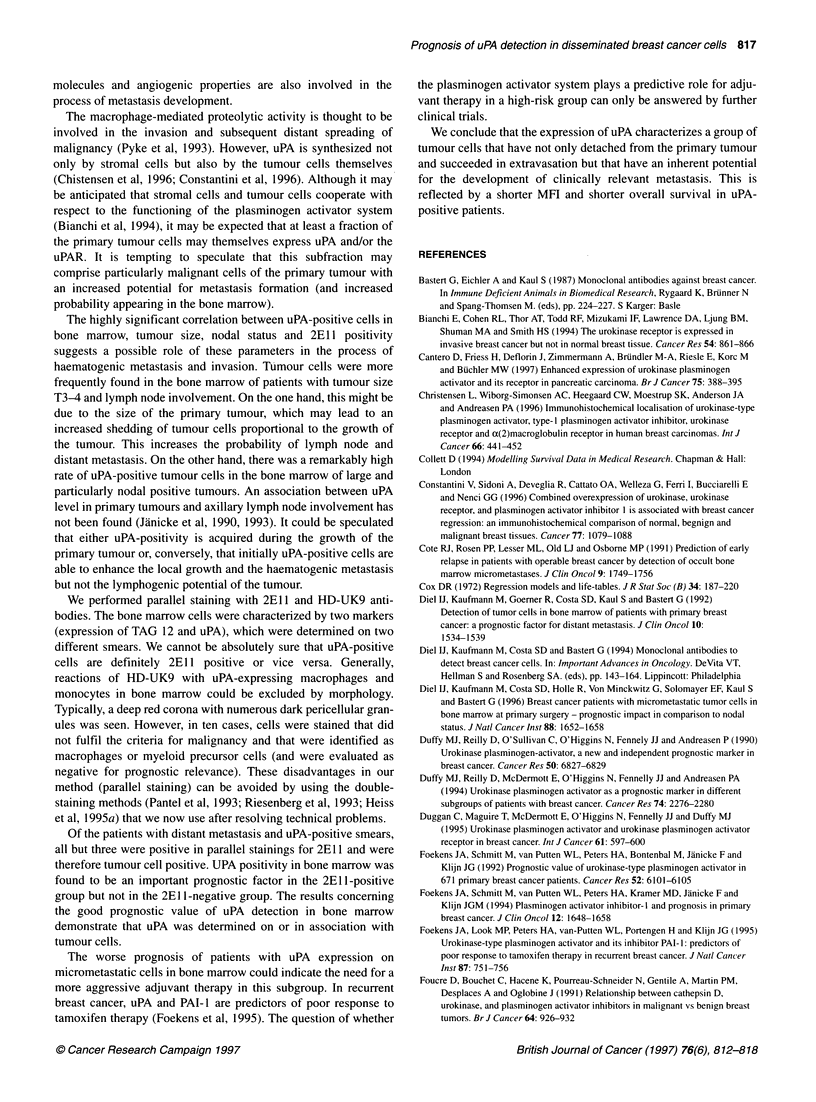

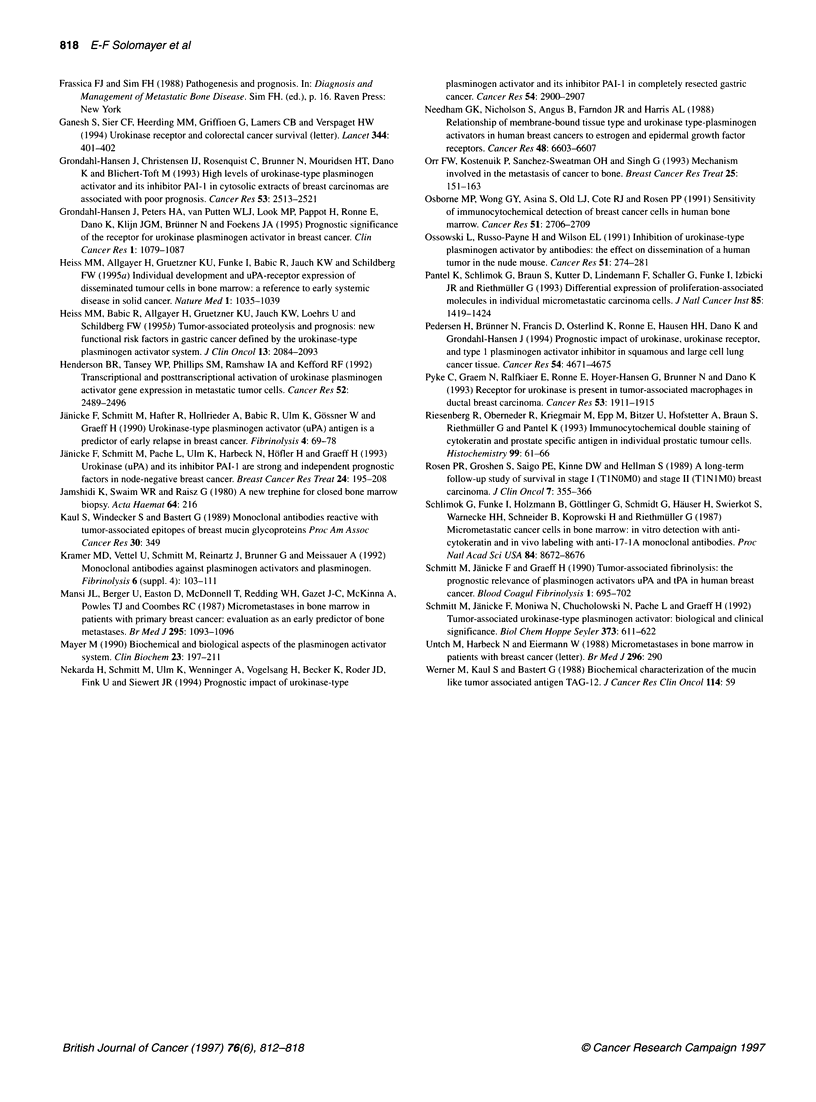


## References

[OCR_00813] Bianchi E., Cohen R. L., Thor A. T., Todd R. F., Mizukami I. F., Lawrence D. A., Ljung B. M., Shuman M. A., Smith H. S. (1994). The urokinase receptor is expressed in invasive breast cancer but not in normal breast tissue.. Cancer Res.

[OCR_00818] Cantero D., Friess H., Deflorin J., Zimmermann A., Bründler M. A., Riesle E., Korc M., Büchler M. W. (1997). Enhanced expression of urokinase plasminogen activator and its receptor in pancreatic carcinoma.. Br J Cancer.

[OCR_00823] Christensen L., Wiborg Simonsen A. C., Heegaard C. W., Moestrup S. K., Andersen J. A., Andreasen P. A. (1996). Immunohistochemical localization of urokinase-type plasminogen activator, type-1 plasminogen-activator inhibitor, urokinase receptor and alpha(2)-macroglobulin receptor in human breast carcinomas.. Int J Cancer.

[OCR_00833] Costantini V., Sidoni A., Deveglia R., Cazzato O. A., Bellezza G., Ferri I., Bucciarelli E., Nenci G. G. (1996). Combined overexpression of urokinase, urokinase receptor, and plasminogen activator inhibitor-1 is associated with breast cancer progression: an immunohistochemical comparison of normal, benign, and malignant breast tissues.. Cancer.

[OCR_00843] Cote R. J., Rosen P. P., Lesser M. L., Old L. J., Osborne M. P. (1991). Prediction of early relapse in patients with operable breast cancer by detection of occult bone marrow micrometastases.. J Clin Oncol.

[OCR_00855] Diel I. J., Kaufmann M., Costa S. D., Bastert G. (1994). Monoclonal antibodies to detect breast cancer cells in bone marrow.. Important Adv Oncol.

[OCR_00860] Diel I. J., Kaufmann M., Costa S. D., Holle R., von Minckwitz G., Solomayer E. F., Kaul S., Bastert G. (1996). Micrometastatic breast cancer cells in bone marrow at primary surgery: prognostic value in comparison with nodal status.. J Natl Cancer Inst.

[OCR_00849] Diel I. J., Kaufmann M., Goerner R., Costa S. D., Kaul S., Bastert G. (1992). Detection of tumor cells in bone marrow of patients with primary breast cancer: a prognostic factor for distant metastasis.. J Clin Oncol.

[OCR_00871] Duffy M. J., Reilly D., McDermott E., O'Higgins N., Fennelly J. J., Andreasen P. A. (1994). Urokinase plasminogen activator as a prognostic marker in different subgroups of patients with breast cancer.. Cancer.

[OCR_00866] Duffy M. J., Reilly D., O'Sullivan C., O'Higgins N., Fennelly J. J., Andreasen P. (1990). Urokinase-plasminogen activator, a new and independent prognostic marker in breast cancer.. Cancer Res.

[OCR_00876] Duggan C., Maguire T., McDermott E., O'Higgins N., Fennelly J. J., Duffy M. J. (1995). Urokinase plasminogen activator and urokinase plasminogen activator receptor in breast cancer.. Int J Cancer.

[OCR_00891] Foekens J. A., Look M. P., Peters H. A., van Putten W. L., Portengen H., Klijn J. G. (1995). Urokinase-type plasminogen activator and its inhibitor PAI-1: predictors of poor response to tamoxifen therapy in recurrent breast cancer.. J Natl Cancer Inst.

[OCR_00881] Foekens J. A., Schmitt M., van Putten W. L., Peters H. A., Bontenbal M., Jänicke F., Klijn J. G. (1992). Prognostic value of urokinase-type plasminogen activator in 671 primary breast cancer patients.. Cancer Res.

[OCR_00886] Foekens J. A., Schmitt M., van Putten W. L., Peters H. A., Kramer M. D., Jänicke F., Klijn J. G. (1994). Plasminogen activator inhibitor-1 and prognosis in primary breast cancer.. J Clin Oncol.

[OCR_00898] Foucré D., Bouchet C., Hacène K., Pourreau-Schneider N., Gentile A., Martin P. M., Desplaces A., Oglobine J. (1991). Relationship between cathepsin D, urokinase, and plasminogen activator inhibitors in malignant vs benign breast tumours.. Br J Cancer.

[OCR_00914] Ganesh S., Sier C. F., Heerding M. M., Griffioen G., Lamers C. B., Verspaget H. W. (1994). Urokinase receptor and colorectal cancer survival.. Lancet.

[OCR_00919] Grøndahl-Hansen J., Christensen I. J., Rosenquist C., Brünner N., Mouridsen H. T., Danø K., Blichert-Toft M. (1993). High levels of urokinase-type plasminogen activator and its inhibitor PAI-1 in cytosolic extracts of breast carcinomas are associated with poor prognosis.. Cancer Res.

[OCR_00926] Grøndahl-Hansen J., Peters H. A., van Putten W. L., Look M. P., Pappot H., Rønne E., Dano K., Klijn J. G., Brünner N., Foekens J. A. (1995). Prognostic significance of the receptor for urokinase plasminogen activator in breast cancer.. Clin Cancer Res.

[OCR_00939] Heiss M. M., Babic R., Allgayer H., Gruetzner K. U., Jauch K. W., Loehrs U., Schildberg F. W. (1995). Tumor-associated proteolysis and prognosis: new functional risk factors in gastric cancer defined by the urokinase-type plasminogen activator system.. J Clin Oncol.

[OCR_00945] Henderson B. R., Tansey W. P., Phillips S. M., Ramshaw I. A., Kefford R. F. (1992). Transcriptional and posttranscriptional activation of urokinase plasminogen activator gene expression in metastatic tumor cells.. Cancer Res.

[OCR_00956] Jänicke F., Schmitt M., Pache L., Ulm K., Harbeck N., Höfler H., Graeff H. (1993). Urokinase (uPA) and its inhibitor PAI-1 are strong and independent prognostic factors in node-negative breast cancer.. Breast Cancer Res Treat.

[OCR_00961] Landys K. (1980). A new trephine for closed bone marrow biopsy.. Acta Haematol.

[OCR_00975] Mansi J. L., Berger U., Easton D., McDonnell T., Redding W. H., Gazet J. C., McKinna A., Powles T. J., Coombes R. C. (1987). Micrometastases in bone marrow in patients with primary breast cancer: evaluation as an early predictor of bone metastases.. Br Med J (Clin Res Ed).

[OCR_00982] Mayer M. (1990). Biochemical and biological aspects of the plasminogen activation system.. Clin Biochem.

[OCR_00993] Needham G. K., Nicholson S., Angus B., Farndon J. R., Harris A. L. (1988). Relationship of membrane-bound tissue type and urokinase type plasminogen activators in human breast cancers to estrogen and epidermal growth factor receptors.. Cancer Res.

[OCR_00986] Nekarda H., Schmitt M., Ulm K., Wenninger A., Vogelsang H., Becker K., Roder J. D., Fink U., Siewert J. R. (1994). Prognostic impact of urokinase-type plasminogen activator and its inhibitor PAI-1 in completely resected gastric cancer.. Cancer Res.

[OCR_00999] Orr F. W., Kostenuik P., Sanchez-Sweatman O. H., Singh G. (1993). Mechanisms involved in the metastasis of cancer to bone.. Breast Cancer Res Treat.

[OCR_01004] Osborne M. P., Wong G. Y., Asina S., Old L. J., Cote R. J., Rosen P. P. (1991). Sensitivity of immunocytochemical detection of breast cancer cells in human bone marrow.. Cancer Res.

[OCR_01009] Ossowski L., Russo-Payne H., Wilson E. L. (1991). Inhibition of urokinase-type plasminogen activator by antibodies: the effect on dissemination of a human tumor in the nude mouse.. Cancer Res.

[OCR_01014] Pantel K., Schlimok G., Braun S., Kutter D., Lindemann F., Schaller G., Funke I., Izbicki J. R., Riethmüller G. (1993). Differential expression of proliferation-associated molecules in individual micrometastatic carcinoma cells.. J Natl Cancer Inst.

[OCR_01020] Pedersen H., Brünner N., Francis D., Osterlind K., Rønne E., Hansen H. H., Danø K., Grøndahl-Hansen J. (1994). Prognostic impact of urokinase, urokinase receptor, and type 1 plasminogen activator inhibitor in squamous and large cell lung cancer tissue.. Cancer Res.

[OCR_01026] Pyke C., Graem N., Ralfkiaer E., Rønne E., Høyer-Hansen G., Brünner N., Danø K. (1993). Receptor for urokinase is present in tumor-associated macrophages in ductal breast carcinoma.. Cancer Res.

[OCR_01031] Riesenberg R., Oberneder R., Kriegmair M., Epp M., Bitzer U., Hofstetter A., Braun S., Riethmüller G., Pantel K. (1993). Immunocytochemical double staining of cytokeratin and prostate specific antigen in individual prostatic tumour cells.. Histochemistry.

[OCR_01037] Rosen P. R., Groshen S., Saigo P. E., Kinne D. W., Hellman S. (1989). A long-term follow-up study of survival in stage I (T1N0M0) and stage II (T1N1M0) breast carcinoma.. J Clin Oncol.

[OCR_01042] Schlimok G., Funke I., Holzmann B., Göttlinger G., Schmidt G., Häuser H., Swierkot S., Warnecke H. H., Schneider B., Koprowski H. (1987). Micrometastatic cancer cells in bone marrow: in vitro detection with anti-cytokeratin and in vivo labeling with anti-17-1A monoclonal antibodies.. Proc Natl Acad Sci U S A.

[OCR_01051] Schmitt M., Jänicke F., Graeff H. (1990). Tumour-associated fibrinolysis: the prognostic relevance of plasminogen activators uPA and tPA in human breast cancer.. Blood Coagul Fibrinolysis.

[OCR_01056] Schmitt M., Jänicke F., Moniwa N., Chucholowski N., Pache L., Graeff H. (1992). Tumor-associated urokinase-type plasminogen activator: biological and clinical significance.. Biol Chem Hoppe Seyler.

[OCR_01065] Seifert J., Machková Z. (1988). Biosynthesis of pyrimidine nucleotides and level of cytochrome P-450 in rat liver and kidney after clofibrate administration (an in vivo study).. J Cancer Res Clin Oncol.

[OCR_01061] Untch M., Harbeck N., Eiermann W. (1988). Micrometastases in bone marrow in patients with breast cancer.. Br Med J (Clin Res Ed).

